# Pharmacokinetics and pharmacogenomics of clozapine in an ancestrally diverse sample: a longitudinal analysis and genome-wide association study using UK clinical monitoring data

**DOI:** 10.1016/S2215-0366(23)00002-0

**Published:** 2023-03

**Authors:** Antonio F Pardiñas, Djenifer B Kappel, Milly Roberts, Francesca Tipple, Lisa M Shitomi-Jones, Adrian King, John Jansen, Marinka Helthuis, Michael J Owen, Michael C O'Donovan, James T R Walters

**Affiliations:** a MRC Centre for Neuropsychiatric Genetics and Genomics, Division of Psychological Medicine and Clinical Neurosciences, School of Medicine, Cardiff University, Cardiff, UK; b Magna Laboratories Ltd, Ross-on-Wye, UK; c Leyden Delta BV, Nijmegen, the Netherlands

## Abstract

**Background:**

The antipsychotic, clozapine, is the only licensed drug against the treatment-resistant symptoms that affect 20–30% of people with schizophrenia. Clozapine is markedly underprescribed, partly because of concerns about its narrow therapeutic range and adverse drug reaction profile. Both concerns are linked to drug metabolism, which varies across populations globally and is partly genetically determined. Our study aimed to use a cross-ancestry genome-wide association study (GWAS) design to investigate variations in clozapine metabolism within and between genetically inferred ancestral backgrounds, to discover genomic associations to clozapine plasma concentrations, and to assess the effects of pharmacogenomic predictors across different ancestries.

**Methods:**

In this GWAS, we analysed data from the UK Zaponex Treatment Access System clozapine monitoring service as part of the CLOZUK study. We included all available individuals with clozapine pharmacokinetic assays requested by their clinicians. We excluded people younger than 18 years, or whose records contained clerical errors, or with blood drawn 6–24 h after dose, a clozapine or norclozapine concentration less than 50 ng/mL, a clozapine concentration of more than 2000 ng/mL, a clozapine-to-norclozapine ratio outside of the 0·5–3·0 interval, or a clozapine dose of more than 900 mg/day. Using genomic information, we identified five biogeographical ancestries: European, sub-Saharan African, north African, southwest Asian, and east Asian. We did pharmacokinetic modelling, a GWAS, and a polygenic risk score association analysis using longitudinal regression analysis with three primary outcome variables: two metabolite plasma concentrations (clozapine and norclozapine) and the clozapine-to-norclozapine ratio.

**Findings:**

19 096 pharmacokinetic assays were available for 4760 individuals in the CLOZUK study. After data quality control, 4495 individuals (3268 [72·7%] male and 1227 [27·3%] female; mean age 42·19 years [range 18–85]) linked to 16 068 assays were included in this study. We found a faster average clozapine metabolism in people of sub-Saharan African ancestry than in those of European ancestry. By contrast, individuals with east Asian or southwest Asian ancestry were more likely to be slow clozapine metabolisers than those with European ancestry. Eight pharmacogenomic loci were identified in the GWAS, seven with significant effects in non-European groups. Polygenic scores generated from these loci were associated with clozapine outcome variables in the whole sample and within individual ancestries; the maximum variance explained was 7·26% for the metabolic ratio.

**Interpretation:**

Longitudinal cross-ancestry GWAS can discover pharmacogenomic markers of clozapine metabolism that, individually or as polygenic scores, have consistent effects across ancestries. Our findings suggest that ancestral differences in clozapine metabolism could be considered for optimising clozapine prescription protocols for diverse populations.

**Funding:**

UK Academy of Medical Sciences, UK Medical Research Council, and European Commission.

## Introduction

Schizophrenia is typically a chronic disorder with symptoms that are severe and distressing for most people. Most people with schizophrenia obtain some benefit from antipsychotic medication,^1^ although about 20–30% experience treatment-resistant schizophrenia, defined as the persistence of symptoms after a minimum of two trials of antipsychotics of adequate dose and duration.^2^ Clozapine is the only approved and evidence-based drug treatment for treatment-resistant schizophrenia and is associated with increased adherence to treatment, decreased psychiatric hospital admission, reduced suicidal ideation, and improvement of symptoms and outcomes.^3^, ^4^, ^5^ Despite its potential, clozapine is not always prescribed to those who could benefit from it. Retrospective analyses have shown that clozapine accounted for less than 5% of all antipsychotics prescribed for schizophrenia during 2006–2009 in the USA,^6^ and that only a third of people eligible in the UK in 2019 were estimated to have received it.^7^ Some important factors that prevent increased clozapine use are insufficient clinician training or direct experience of using clozapine, and concerns about its adverse drug reaction profile.^8^

**Research in context**

**Evidence before this study**

The latest reviews of clozapine prescription guidelines, published in March and June, 2022, recommend different dosing regimens for some ethnicity and population groups, owing to differential drug metabolisms. People of east Asian ancestry and Indigenous people of the Americas were identified as slow clozapine metabolisers and were advised to be prescribed lower clozapine maintenance doses than for people of European or west Asian ancestry. No recommendations were made for people with other ancestries, although the reviewed evidence was recognised as “limited”. We searched MEDLINE, Embase, and the Cochrane Library on Sept 12, 2022, using the search terms “clozapine” AND (“guide*” OR “recommendation”), for papers published in English from Jan 1, 2020, to Sept 12, 2022, and found no primary data beyond that included in the reviews. No genetic variants associated with clozapine metabolism were considered of clinical utility in the reviews of clozapine dosing guidelines. We also searched the Pharmacogenomics Knowledgebase (PharmGKB) on Sept 12, 2022, and found no dosing recommendations informed by pharmacogenomics for clozapine. PharmGKB indexes all reports by the Clinical Pharmacogenetics Implementation Consortium, the Royal Dutch Pharmacogenetics Working Group, the Canadian Pharmacogenomics Network for Drug Safety, and the French National Network of Pharmacogenetics.

**Added value of this study**

In this study, we found ancestral effects and genetic variants associated with inter-individual variation in clozapine metabolism. We found that people of sub-Saharan African ancestry were on average fast clozapine metabolisers, and that those of east Asian or southwest Asian ancestry were slow clozapine metabolisers, all in comparison with people of European ancestry. We also showed that genomic predictors of clozapine pharmacokinetics, developed using longitudinal genome-wide association study and polygenic risk score approaches, had associations of similar effects across diverse ancestral backgrounds.

**Implications of all the available evidence**

Existing clozapine treatment guidelines do not reflect the real-world diversity of potential clozapine users, and default recommendations for titration and maintenance doses are optimised for those of European descent. Our study reinforces that ancestry can contribute to the variability of clozapine metabolism, and it supports the proactive assessment of drug pharmacokinetics (via therapeutic drug monitoring) as part of the standard care for those with treatment-resistant schizophrenia. For the development of further research on this topic, our data support the view that pharmacogenomic predictors of clozapine metabolism might be applicable across worldwide populations, which could facilitate the design and implementation of prospective trials to assess the incorporation of genomics to personalised clozapine prescribing.

Health services in many countries mandate haematological monitoring for people receiving clozapine, to prevent the rare but potentially fatal outcome of agranulocytosis, and many other potential adverse reactions are also challenging for clinical management.^9^ Some of these reactions, including paralytic ileus and seizures, can be life-threatening and are associated with clozapine metabolism, a complex interplay of biological processes that can also have a role in treatment outcomes.^10^ To balance efficacy and the adverse drug reaction risks, guidelines recommend to prescribe clozapine maintenance doses that lead to plasma concentrations of 350–600 ng/mL, which is sometimes referred to as the therapeutic range.^11^ However, without regular assessments of clozapine pharmacokinetics (a framework known as therapeutic drug monitoring), the large inter-individual variability in clozapine metabolism makes optimal prescription of safe and efficacious doses of clozapine a trial-and-error exercise.^10^ This uncertainty persists even after accounting for robust predictors of drug metabolism (eg, smoking, co-medications, and bodyweight), which can explain up to 50% of the variance in clozapine plasma concentrations in retrospective studies.^12^

Pharmacogenomic approaches are established experimental designs for discovering novel predictors of drug metabolism and treatment response, and they can often highlight specific genetic markers among well known biological pathways and enzyme mechanisms.^13^ Genome-wide association studies (GWASs) have pointed towards a small number of variants that account for an additional 1–10% of the variance in clozapine pharmacokinetics.^14^, ^15^ These studies have only been done in individuals of European ancestry. This fact is a clear limitation, given the known diversity of drug-metabolising enzyme alleles globally, and potentially leads to genomic predictors that are not transferable across populations.^13^ The Eurocentrism of clozapine pharmacogenomics studies is particularly problematic because the prescription patterns and outcomes of this drug seem to be ancestrally stratified to some extent. For example, the titration and maintenance doses recommended for people of east Asian ancestry and Indigenous people of the Americas are lower than doses typically prescribed to people of European ancestry, to compensate for a generally slower clozapine metabolism in these populations.^16^, ^17^ The importance of considering ancestral background in optimal clozapine prescribing, particularly in pharmacogenomics research, needs to be thoroughly investigated in large and ancestrally diverse samples. However, recruiting individuals for genomic studies, either internationally or from populations that form minorities in Europe and North America, is a complex endeavour because of the impairments and poor health caused by treatment-resistant schizophrenia, which act alongside a larger set of social and cultural barriers influencing participation and trust in clinical and academic schizophrenia research.^18^ In this study, we sought to explore the ancestral diversity of common genetic variation relevant for clozapine metabolism, by developing statistical models of clozapine pharmacokinetics and doing a GWAS on five ancestral biogeographical groups (people with European, sub-Saharan African, north African, southwest Asian, and east Asian ancestry). We also aimed to validate the use of our GWAS approach for deriving genomic predictors of clozapine metabolism.

## Methods

### Study design and samples

In this GWAS, we did a cross-ancestry analysis of clozapine metabolism in individuals linked to genomic data and longitudinal blood monitoring assays. Pharmacokinetic and genomic data from the study participants were acquired as part of the CLOZUK study^14^ of individuals from the UK who had been prescribed clozapine for treatment-resistant schizophrenia. The current study involved people included in the Zaponex Treatment Access System, a clozapine monitoring service managed by the pharmaceutical company Leyden Delta (Nijmegen, Netherlands). Samples and data from individuals were collected during the CLOZUK study's second wave (CLOZUK2; 2013–15) and third wave (CLOZUK3; 2019–21). Data from people in CLOZUK2 of European ancestry were reported previously as part of genome-wide analyses of schizophrenia and clozapine metabolism,^4^ and data from CLOZUK3 and from people of non-European ancestry in CLOZUK2 were reported in a study of clozapine prescription patterns.^19^ The CLOZUK study received UK National Research Ethics Service approval (reference 10/WSE02/15), in accordance with the requirements of the UK Human Tissue Act 2004.

### Measures

We included all individuals in CLOZUK with clozapine pharmacokinetic assays, including information on the clozapine and norclozapine plasma concentrations, daily clozapine dose, and the time of both the drug intake and blood draw. Plasma concentrations were determined by a standard high-performance liquid chromatography mass spectrometry procedure at Magna Laboratories (Ross-on-Wye). We excluded data from people whose records contained clerical errors, or with blood drawn 6–24 h after dose, a clozapine or norclozapine concentration less than 50 ng/mL (outside the minimum detection range of the high-performance liquid chromatography mass spectrometry instrument), a clozapine concentration more than 2000 ng/mL (ie, reaching the range of potential toxicity), a clozapine-to-norclozapine ratio outside of the 0·5–3·0 interval (ie, indicating non-adherence), or a clozapine dose of more than 900 mg/day (the maximum advised by the British National Formulary). Owing to potentially different treatment regimens, we also excluded individuals younger than 18 years. For further details on our use of the CLOZUK pharmacokinetic data see appendix 1 (p 2).

### Genotyping: quality control and imputation

The CLOZUK2 samples underwent genotyping, quality control, and imputation as described in a previous publication and in appendix 1 (pp 2–3).^14^ The CLOZUK2 samples were genotyped by deCODE Genetics (Reykjavík, Iceland) and the CLOZUK3 samples were genotyped at the Icahn School of Medicine at Mount Sinai (New York City, NY, USA). Genotype data from CLOZUK2 and CLOZUK3 were excluded according to cutoffs on the rate of missingness (>2% for all samples and markers) and inbreeding coefficient (F>0·2 for all samples). Genotype imputation for both cohorts was done by use of the Haplotype Reference Consortium panel through the Michigan Imputation Server. All imputed genotype dosages were further curated within each cohort with various parameters (imputation quality r^2^ ≥0·7; hard-call genotype probability ≥80%; hard-call missingness ≤5%; minor allele count ≥2), and the remaining variants were then merged and curated again (for hard-call missingness ≤2%; minor allele count ≥ 400 and Hardy-Weinberg equilibrium mid p>10^−6^).

### Statistical analysis

For the pharmacokinetic data analysis, generalised linear mixed-effect model (GLMM) regression was used to assess and evaluate differences between ancestral groups in clozapine dosing and metabolism. We analysed five ancestral biogeographical groups, representing people with European, sub-Saharan African, north African, southwest Asian, and east Asian ancestries. Outcome variables were defined as follows: two plasma concentrations in their raw scale (clozapine and its main metabolite N-desmethylclozapine, also known as norclozapine), the logarithm of the clozapine-to-norclozapine metabolic ratio, the logarithm of clozapine doses, and the bins of clozapine plasma concentrations, (defined by whether subtherapeutic [<350 ng/mL], therapeutic [350–600 ng/mL], or supratherapeutic [>600 ng/mL] concentrations were reached).

Regression models for these outcomes were fitted with functions included in the glmmTMB^20^ and ordinal^21^ R packages, assuming gamma probability distributions for the metabolites and normal distributions for the logarithms,^14^ and a cumulative link function for the bins of clozapine plasma concentrations.^21^ In all regressions, fixed-effect covariates included the ancestry classification, the time between dose intake and blood sample, sex, age, and age^2^. Clozapine dose was also included as a fixed-effect predictor in the clozapine, norclozapine, and metabolic ratio regressions owing to its strong linear correlation with these metrics.^12^ One random-effect predictor was fitted at the level of study participants in all models, which allowed all the curated pharmacokinetic assays to be included while preventing confounding owing to repeated measurements (known as pseudoreplication). Individuals classified as being of admixed or unknown biogeographical ancestry were not included in these analyses, because of their potentially heterogeneous genetic makeup.

For the genomic data analyses, we did a GWAS of the clozapine and norclozapine plasma concentrations, and the metabolic ratio, using TrajGWAS (version 0.13; https://github.com/OpenMendel/TrajGWAS.jl),^22^ which used a GLMM framework to model longitudinal measurements in each analysis. This method is an advance on previous approaches that required representative single phenotypes for each individual to be derived from the repeated measures,^14^, ^15^, ^23^ because such constructs might not capture all the relevant variability of the data.^24^ TrajGWAS models included all individuals from CLOZUK2 and CLOZUK3, and used clozapine doses, time between dose intake and blood sample, sex, age, and age^2^ as covariates. As with other genomic association tests based on linear regression, ten genetic principal components were included to control potential population stratification, and four genetic ancestry probabilities were included in place of the categorical ancestry variable. The genome-wide significance level was set, as commonly defined in human genomics, at p≤5 × 10^−8^. Further details on fitting the TrajGWAS regression models and on the power and use of this method are in appendix 1 (pp 4–6). To identify credible causal single nucleotide polymorphisms (SNPs) and genes, we applied statistical fine-mapping to each of the genome-wide significant loci, as described in appendix 1 (p 6). To provide an estimate of the phenotypic effects of those pharmacogenomic variants, we fitted GLMMs to the SNPs with the largest posterior probability of being causal variants at each phenotype and locus combination, again using glmmTMB^20^ in R.

To create independent training and testing sets for polygenic risk score (PRS) analyses, the combined CLOZUK dataset was split in the CLOZUK2 and CLOZUK3 waves. TrajGWAS was run by use of CLOZUK2 data with identical models and parameters to the main analyses to generate training summary statistics. PRSice-2 (version 2.35; https://choishingwan.github.io/PRSice/)^25^ was then applied to CLOZUK3 data to generate clozapine, norclozapine, and metabolic ratio PRS on the basis of the CLOZUK2 results. To assess the effect of polygenicity in the PRS association analyses, nine different SNP p value inclusion thresholds were used to generate PRSs: p<5 × 10^−8^, p<1 × 10^−5^, p<1 × 10^−4^, p<0·001, p<0·01, p<0·05, p<0·1, p<0·5, and p<1·0.

For PRS regression we used GLMMs in R analogously to the pharmacokinetic analyses, using the full CLOZUK3 longitudinal dataset. For consistency with our GWAS approach, each GLMM assessing the effects of a single PRS incorporated as fixed-effect covariates the clozapine daily dose, time between dose intake and blood sample, sex, age, age^2^, ten principal components, and four genetic ancestry probabilities. A random-effect covariate was also introduced at the participant level. To account for multiplicity of tests throughout this procedure, PRS-association p values were corrected using false discovery rates^26^ within each outcome and were considered significant at a threshold ofp_false discovery rate_≤0·05. An index of variance explained for each PRS, independently of other fixed-effect and random-effect covariates, was also computed (appendix 1 p 6).

### Role of the funding source

The funder of the study had no role in study design, data collection, data analysis, data interpretation, or writing of the report.

## Results

19 096 pharmacokinetic assays were available for 4760 CLOZUK individuals. After exclusions, 4495 individuals (3268 [72·7%] male and 1227 [27·3%] female; mean age 42·19 years [range 18–85]) linked to 16 068 pharmacokinetic assays remained in the combined CLOZUK2 and CLOZUK3 dataset. The first assay was recorded on Oct 31, 2011, and the most recent on Oct 29, 2018. A sample size breakdown by genetic ancestry and data collection wave is shown in table 1. A detailed report on clozapine pharmacokinetics and doses for all genetic ancestry groups is in appendix 2 (sheet 2).

**Table 1:** Individuals and clozapine pharmacokinetic assays, by data collection wave and genetic ancestry group

	**CLOZUK2 dataset**		**CLOZUK3 dataset**	
	Individuals (n=2578)	Assays (n=11 407)	Individuals (n=917)	Assays (n=4661)
European	2910 (81·3%)	9213 (80·8%)	767 (83·6%)	3834 (82·3%)
Sub-Saharan African	192 (5·4%)	598 (5·2%)	51 (5·6%)	242 (5·2%)
North African	108 (3%)	318 (2·8%)	14 (1·5%)	79 (1·7%)
Southwest Asian	200 (5·6%)	706 (6·2%)	44 (4·8%)	237 (5·1%)
East Asian	36 (1·0%)	112 (1·0%)	5 (0·5%)	18 (0·4%)
Admixed or unknown	132 (3·7%)	460 (4·0%)	36 (3·9%)	251 (5·4%)

Data are n (%). Data were collected in 2013–15 for the CLOZUK2 dataset and in 2019–21 for the CLOZUK3 dataset.

Regarding ancestral differences in clozapine pharmacokinetics, significant differences (p<0·05) were observed for most of the pharmacokinetic comparisons between the European ancestry and the four non-European ancestry groups (table 2). All non-European ancestries showed a slightly increased but significant clozapine-to-norclozapine metabolic ratio compared with Europeans, whereas the results for individual clozapine metabolites varied between ancestries.

**Table 2:** Estimated ancestral differences in clozapine pharmacokinetic outcomes, by genetic ancestry group

	**Log (dose)**				**Clozapine**				**Norclozapine**				**Log (metabolic ratio)**			
	Relative percentage change	Estimated effect size	SE	p value	Relative percentage change	Estimated effect size	SE	p value	Relative percentage change	Estimated effect size	SE	p value	Relative percentage change	Estimated effect size	SE	p value
European (n=3677)	Ref	Ref	..	..	Ref	Ref	..	..	Ref	Ref	..	..	Ref	Ref	..	..
Sub-Saharan African (n=243)	−0·67%	−0·007	0·026	0·80	−8·52%	−0·088	0·034	0·010	−24·40%	−0·280	0·032	2·21 × 10^−18^	26·64%	0·191	0·017	1·73 × 10^−29^
North African (n=122)	−12·32%	−0·132	0·037	3·67 × 10^−4^	4·81%	0·045	0·048	0·35	−3·03%	−0·025	0·045	0·59	10·04%	0·072	0·024	0·0026
Southwest Asian (n=244)	−14·84%	−0·161	0·026	6·31 × 10^−10^	14·44%	0·135	0·034	5·91 × 10^−5^	7·57%	0·076	0·031	0·015	8·51%	0·061	0·017	2·67 × 10^−4^
East Asian (n=41)	−14·95%	−0·162	0·063	0·010	17·41%	0·162	0·082	0·049	5·30%	0·055	0·077	0·47	15·76%	0·113	0·041	0·0056

Data are %, estimated effect size, or SE. Effect sizes were estimated by use of generalised linear mixed model regression. Estimated effect sizes indicate whether individuals within each ancestry group have a greater (positive) or lower (negative) average outcome value than those of European ancestry; the reference value was individuals of European ancestry, because they comprised the largest sample size in the CLOZUK database. For the effect sizes of additional fixed-effect covariates of each model, see appendix 1 (p 9).

**Figure 1:**
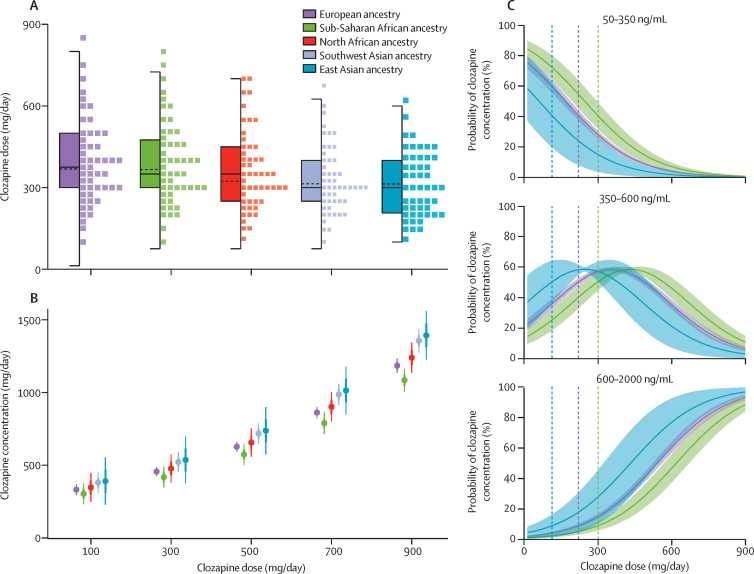
Analysis of clozapine pharmacokinetics in the CLOZUK longitudinal dataset, by ancestry group (A) Distribution of clozapine doses throughout treatment, stratified by genetic ancestry group; lines at the centre of the boxplots indicate median (solid line) or mean (dotted line) clozapine doses and boxes represent the IQR. (B) Marginal effects, or effect sizes independent of other covariates in the model, of the ancestry groups in the relationship between clozapine doses and plasma concentrations with mean concentrations at specific doses (dots), 95% CIs (boxes), and full ranges (bar lines). (C) Marginal effects of the east Asian, European, sub-Saharan African ancestry groups in the relationship between clozapine doses and the probability (line) and 95% CI (shaded area) of reaching clozapine concentrations inside or outside the therapeutic range (350–600 ng/mL). Vertical dashed bars show the doses required by individuals in each ancestry group have a 50% probability of reaching the therapeutic range. Individuals with sub-Saharan ancestry required doses of 300 mg/day to reach the therapeutic interval with at least 50% probability (50·05%; SE 2·86%), whereas this outcome was achieved at 220 mg/day in people of European ancestry (50·23%; SE 1·22%) and at 112 mg/day in people of east Asian ancestry (50·32%; SE 6·93%). The north African and southwest Asian groups are not shown here to avoid overplotting as their probability curves largely overlap those of other ancestries; a complete version is in appendix 1 (p 18).

GLMM effect sizes, controlled for dose and the other factors listed earlier, pointed towards slower clozapine metabolism in people with Asian ancestry than in those with European ancestry, as indexed by higher average clozapine plasma concentrations in those of east Asian (estimated effect size [β] 0·162; standard error [SE] 0·082; p=0·049) and southwest Asian (β 0·135; SE 0·034; p=5·91 × 10^−5^) ancestry. Compared with people of European ancestry, norclozapine plasma concentrations were also significantly higher in people with southwest Asian ancestry (β 0·076; SE 0·031; p=0·015), an effect probably mediated by their higher clozapine concentrations and which disappeared after controlling for clozapine plasma concentrations in this analysis (β –0·008; SE 0·018; p=0·676). Consistent with these observations, in analyses of the clozapine doses prescribed throughout treatment, people of east and southwest Asian ancestry typically received lower doses of clozapine than did those of Europeans ancestry (figure 1A). These differences in plasma concentrations and doses had a similar magnitude to the effects of sex (appendix 1 p 9).

Individuals of sub-Saharan African ancestry showed lower plasma concentrations than those with European ancestry for clozapine (β –0·088; SE 0·034; p=0·010) and norclozapine (β –0·280; SE 0·032; p=2·21 × 10^−18^), pointing towards faster metabolism (figure 1B). There were no significant differences in average clozapine doses between those of sub-Saharan African and European ancestries (β –0·007; SE 0·026; p=0·80).

To assess indirectly whether differences in prescriptions and metabolism might have implications for the effectiveness of clozapine, we used ordinal GLMMs to assess the probability of individuals of each ancestry having clozapine plasma concentrations within, below, or above the therapeutic range (350–600 ng/mL). Results of this model showed that people of southwest Asian ancestry were approximately twice as likely as those of European ancestry to reach therapeutic or supratherapeutic clozapine concentrations throughout their treatment (odds ratio [OR] 1·974; SE 0·189; p=3·29 × 10^−4^), whereas people with sub-Saharan African ancestry were half as likely as those with European ancestry (OR 0·560; SE 0·193; p=2·67 × 10^−3^; appendix 2, sheet 6). Probability estimates of specific plasma concentration categories as a function of the clozapine dose also showed increased likelihood of subtherapeutic concentrations among people of sub-Saharan African ancestry in CLOZUK (figure 1C). Specifically, these individuals required doses of 300 mg/day to reach the therapeutic interval with at least 50% probability (50·05%; SE 2·86%), whereas the same outcome was achieved at 220 mg/day in people of European ancestry (50·23%; SE 1·22%) and at 112 mg/day in those of east Asian ancestry (50·32%; SE 6·93%).

**Figure 2:**
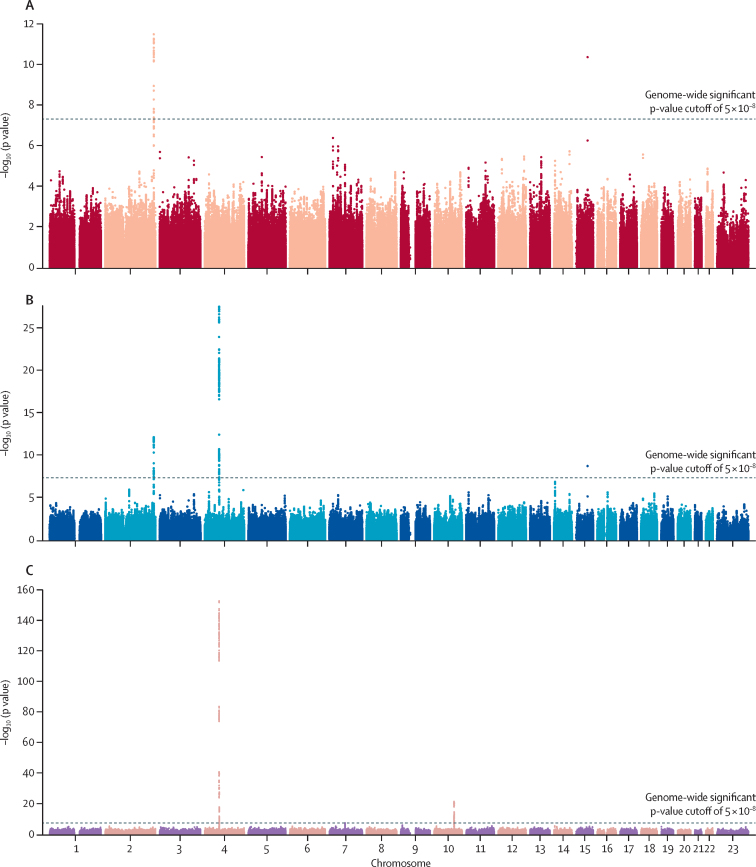
Manhattan plots of the genome-wide association study analyses of clozapine metabolism Colours used do not represent any additional information and are for display purposes only. (A) clozapine plasma concentrations (λ_GC_=1·028). (B) norclozapine plasma concentrations (λ_GC_=1·018). (C) clozapine-to-norclozapine metabolic ratio (λ_GC_=1). λ_GC_=genomic inflation factor.

For the genomic data, in CLOZUK2, 7417 samples were genotyped, and after curation and merging with the pharmacokinetic assay dataset, 3578 samples genotyped at 698 442 SNPs remained. In CLOZUK3, 1439 samples were genotyped and after curation and merging, 917 samples genotyped at 537 334 SNPs remained. After imputation and merging of all genotype data, 2·91 million SNPs were available for analysis. A Manhattan plot for the cross-ancestry GWAS of the plasma concentrations of clozapine and norclozapine, and the clozapine-to-norclozapine metabolic ratio, is in figure 2. Across all phenotypes, eight genome-wide significant loci were found (table 3), five of which have already been reported in people of European ancestry.^14^, ^15^ We noted novel cross-phenotype convergences in two of these known regions: *CYP1A1/1A2* (chromosome 15) and UGT1A* (chromosome 2). These loci were previously associated to clozapine (*CYP1A1*/*1A2*) and norclozapine (UGT1A*), but were associated to both phenotypes in our current analysis. We also found a novel signal for the metabolic ratio, indexed by the SNP rs41301394 (β 0·196, SE 0·035, p=4·81 × 10^−8^), an intronic PharmGKB-annotated variant within *POR*, which is the gene encoding the NADPH–cytochrome P450 oxidoreductase protein. No additional genome-wide significant associations were seen in any of the ancestry-specific GWAS (appendix 1 pp 13–16), although the European-only ancestry analysis supported all our main results except for the locus-tagging *POR*. The *CYP2C18* metabolic ratio association surpassed the statistical threshold for genome-wide significance in the southwest Asian-only analysis.

**Table 3:** Genome-wide significant loci associated with clozapine metabolism

**Phenotype and locus (containing all variants with R^2^>0·1 to index)**	**Index single nucleotide polymorphism**	**p value**	Tagged genes*
**Clozapine**			
chr2:234610912–234676118	rs3732218	3·26 × 10^−^12	*DNAJB3, MROH2A, UGT1A1, UGT1A3, UGT1A4,* UGT1A5, UGT1A6, UGT1A7, UGT1A8, UGT1A9, UGT1A10
chr15:75019449–75027880	rs2472297	4·39 × 10^−^11	CYP1A1, CYP1A2
**Norclozapine**			
chr4:69557365–70093260	rs2926036	3·16 × 10^−^28	*UGT2A3, UGT2B4, UGT2B7,* UGT2B10*, UGT2B11, UGT2B28*
chr2:234610912–234676118	rs3732218	8·07 × 10^−^13	*DNAJB3, MROH2A, UGT1A1, UGT1A3, UGT1A4,* UGT1A5, UGT1A6, UGT1A7, UGT1A8, UGT1A9, UGT1A10
chr15:75019449–75027880	rs2472297	2·10 × 10^−^9	CYP1A1, CYP1A2
**Metabolic ratio**			
chr4:69603602–70366972	rs835309	2·07 × 10^−^153	*UGT2A3, UGT2B4, UGT2B7,* UGT2B10*, UGT2B11, UGT2B28*
chr10:96024357–96848776	rs1926711	4·74 × 10^−^22	*CYP2C8, CYP2C9,* CYP2C18*, CYP2C19, HELLS, NOC3L, PLCE1, TBC1D12*
chr7:75607155–75843524	rs41301394	4·81 × 10^−^8	*MDH2,* POR*, SRRM3, STYXL1, TMEM120A*

* The closest gene to the index single nucleotide polymorphism (in GRCh37 [also known as hg19] coordinates) is shown in bold, with multiple genes shown in bold for overlapping gene boundaries or in intergenic single nucleotide polymorphisms.

After statistical fine-mapping of the genome-wide significant loci, and fitting GLMMs to the SNPs with the largest posterior probability of being causal variants at each phenotype and locus combination, the association statistics were similar to the TrajGWAS results, with all markers retaining genome-wide significance (appendix 1 p 10). Performing this analysis within ancestries showed that all genome-wide significant loci, except POR, had nominally significant effects in at least one non-European biogeographical group (appendix 2 sheet 5).

**Figure 3:**
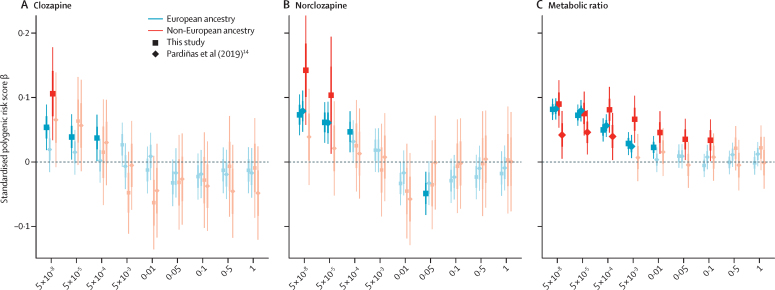
Association between polygenic risk scores for clozapine metabolism generated from a CLOZUK2 genome-wide association study and their corresponding phenotypes in the CLOZUK3 longitudinal dataset Squares and diamonds indicate the estimated value of the regression effect size, all associated bars indicate SEs, and thin lines indicate 95% CIs. Dark colours indicate all significant associations not corrected for multiple testing; corrected p values and all associated effect sizes (pale colours) are in appendix 2 (sheet 2).

In terms of the genomic prediction of clozapine metabolite concentrations in independent datasets, PRS for clozapine and norclozapine plasma concentrations, and their metabolic ratio were generated from the CLOZUK2 data. These scores were associated, at several p value thresholds, with their respective phenotypes in CLOZUK3, explaining a maximum of 0·61% (clozapine), 1·59% (norclozapine), and 7·26% (metabolic ratio) of the variance after accounting for fixed-effect and random-effect predictors (appendix 2 sheet 3). In these analyses, and for all phenotypes, PRSs that showed the stronger association and the greater variance explained were those built only with genome-wide significant SNPs, and all associations at this p value threshold remained significant after splitting the testing dataset by European versus non-European ancestry. We compared this PRS predictor with one that was analogously generated from our 2019 European-only GWAS.^14^ The cross-ancestry PRS predictor showed significant associations at more PRS p value thresholds than the 2019 GWAS PRS predictor did. Furthermore, all PRS p value thresholds that yielded a significant association with the 2019 GWAS PRS were also significant when tested with the cross-ancestry PRS (figure 3). Many of these associations were also replicated with similar effect sizes in the two largest non-European ancestries within CLOZUK3 (ie, the sub-Saharan African and the southwest Asian groups), which supports the transferability of our PRSs as predictors of clozapine metabolism to under-represented populations in genomic research (appendix 1 p 17).

## Discussion

To our knowledge, this study is the first to explore clozapine metabolism and pharmacogenomics using a diverse cross-ancestry sample and statistical methods that take advantage of the longitudinal nature of clozapine monitoring assays. Using pharmacokinetic data we showed that, on average, individuals of sub-Saharan African ancestry were more likely than those of European ancestry to be fast clozapine metabolisers but were not prescribed different doses to them. We also showed that people of sub-Saharan African ancestry were the least likely in our dataset to achieve therapeutic plasma concentrations of clozapine, which parallels findings in non-clozapine antipsychotics.^27^ These results are of potential clinical importance, given that the most recent revisions of clozapine treatment guidelines do not report pharmacokinetic differences between people of European versus African or African American ancestry.^16^, ^17^ Additionally, studies in the USA highlight racial disparities in clozapine use.^18^ Given that the evidence supporting the clozapine therapeutic range being consistently and cross-ancestrally associated with treatment response,^11^ suboptimal prescriptions to people with a different clozapine metabolism rate than what is assumed typical are likely to lead to a lower treatment response and unnecessary exposure to adverse drug reactions. This idea supports the argument that robust assessments of clozapine metabolism, including therapeutic drug monitoring, should be done across ancestries and populations to inform accurate and safe dosing practices for the diverse real-world pool of potential clozapine users.^17^

Our longitudinal cross-ancestry GWAS approach provides evidence that loci previously identified in people of European ancestries also have pharmacokinetic associations in people of other ancestral backgrounds, which is a reassuring finding as some of these variants are only found at low frequencies in most of the world's population and thus are likely to be underpowered for cross-ancestry testing. In particular, the evidence for an association between each of rs3732218 (UGT1A*) and rs2472297 (*CYP1A1/1A2*) with both clozapine and norclozapine plasma concentrations reinforces the view that these represent, or at least index, causal variants influencing pharmacokinetics. However, the congruence in directions of effect of both variants across all analyses suggests that the primary variation in plasma clozapine might be mediating SNP effects on norclozapine concentrations. This outcome would not be unexpected, given that up to 95% of clozapine undergoes demethylation to norclozapine early in its hepatic metabolic pathway, and both compounds can also become secondary or tertiary glucuronides.^13^ Thus, multiple and complex biochemical processes might lead to these pharmacogenomic associations, as the UGT1A* family (particularly *UGT1A4*) has been linked to the excretion of both norclozapine and clozapine,^28^ and *CYP1A2* drives the clozapine to norclozapine conversion and participates in producing clozapine N-oxide,^29^ which is a secondary metabolite that is primarily formed at high concentrations of the drug. Gaining further insight into these associations requires direct experimental validation.

Finally, our polygenic analyses show that pharmacogenomic research studies might benefit from generating PRS by use of a GWAS of drug metabolism. This suggestion is despite the oligogenic genetic architecture of drug pharmacokinetics, seen in many other metabolic traits, instead of the polygenic basis exploited in most PRS-based research. In an oligogenic framework, phenotypic associations with PRS are expected to be stronger at conservative SNP p value thresholds as we observe through our tests,^30^ with the paucity of variants considered in the score being offset by reasonably large per-allele effect sizes (eg, one minor allele of rs2472297 has an effect on clozapine plasma concentrations roughly equivalent to reducing the dose by 50 mg/day^14^). Indeed, we show that all our clozapine metabolism PRSs were associated cross-ancestrally with their respective phenotypes in independent samples, and that their variances explained, despite being small, were consistent with those previously estimated for index SNPs within genome-wide significant loci.^14^ A direct comparison with our previous study^14^ supports the view that in the current study, our novel longitudinal GWAS method and more diverse samples contribute to the increased power of this approach, particularly in those of non-European ancestry, leading to a PRS predictor that is potentially transferrable across ancestries. This approach is promising for future replication and validation studies, as only small panels of SNPs might be needed to evaluate the relevance of these genomic findings for clinical outcomes (eg, treatment response and adverse drug reactions) or prescribing practices, facilitating the design of these studies and the targeting of large and global samples.

This study also had some limitations. The fact that CLOZUK participants were recruited solely within the UK meant that only small sample sizes were available for individuals of non-European ancestries and that these individuals were identified using a genetics-based approach that relied on discrete biogeographical categories. We have attempted to maximise the use of these data by making ancestry-based exclusions only when needed, to avoid potential confounding, and by incorporating as much data as possible into pharmacokinetic and genomic analyses using GLMM regression. However, it is likely that the GWAS we did in people with non-European ancestries was still too underpowered to detect ancestry-specific associations at the genome-wide significant threshold. The absence of information in CLOZUK on treatment adherence behaviour and known predictors of clozapine metabolism (most importantly smoking habits, bodyweight, and concomitant medication) is another limitation of this study. To address this issue, we controlled for treatment adherence by discarding pharmacokinetic assays that reported extreme clozapine metabolic ratios. Also, the main reported effect of not accounting for known exposures in genomic studies of metabolic traits, including clozapine, is the masking of signals.^15^ This issue should nevertheless be considered when evaluating our work in a broader context; it could, for example, downsize the utility or variance explained by SNPs or PRS, particularly if potential mediators (eg, smoking) were increasingly likely in our small non-European subsamples.

In summary, this study adds to the evidence for associations related to clozapine pharmacokinetics, and it establishes cross-ancestral convergences in pharmacogenomic markers. Clozapine dosing and titration protocols that were developed by use of data from populations of European descent are unlikely to be optimal for a substantial proportion of humanity, and current clinical practice should be assisted by therapeutic drug monitoring approaches whenever possible. Our results contribute to the identification of predictors of clozapine metabolism that could be used to design interventions seeking to improve the access and safety of this drug. Our study also shows a benefit of using indexes of genetic ancestry in pharmacological research. As the pharmacogenomics community strives to incorporate diverse populations into its routine work, the future shows promise for the development of personalised medicine initiatives for clozapine treatment.

## Data sharing

GWAS summary statistics are available for download at http://walters.psycm.cf.ac.uk/ and at the National Human Genome Research Institute–European Bioinformatics Institute GWAS Catalog (https://www.ebi.ac.uk/gwas/; accession numbers GCST90239836, GCST90239837, and GCST90239838). To comply with the ethical and regulatory framework of the CLOZUK project, access to individual-level data requires a collaboration agreement with Cardiff University. Requests to access deidentified datasets, data dictionaries, and other summaries from the CLOZUK project should be directed to James T R Walters (waltersjt@cardiff.ac.uk).

For more on the **British National Formulary** see https://bnf.nice.org.uk/

For more on the **Michigan Imputation Server** see http://www.haplotype-reference-consortium.org/

## Declaration of interests
