# Correction to *Lancet Psychiatry* 2023; 10: 209–19

**DOI:** 10.1016/S2215-0366(26)00224-5

**Published:** 2026-09

*Pardiñas AF, Kappel DB, Roberts M, et al. Pharmacokinetics and pharmacogenomics of clozapine in an ancestrally diverse sample: a longitudinal analysis and genome-wide association study using UK clinical monitoring data.* Lancet Psychiatry *2023;* 10: *209–19—*In this Article, participant exclusion criteria should have stated blood drawn less than 6 h or more than 24 h after clozapine dose. This correction was made on July 20, 2026.

