# Supplementary material for: Pharmacokinetics and pharmacogenomics of clozapine in an ancestrally diverse sample: a longitudinal analysis and genome-wide association study using UK clinical monitoring data
Source: Lancet Psychiatry. 2023 Mar;10(3):209–19. doi: 10.1016/S2215-0366(23)00002-0 (PMC10824469; doi:10.1016/S2215-0366(23)00002-0)
Supplement: Supplementary appendix 1 [file mmc1.pdf]

THE LANCET
Psychiatry

Supplementary appendix 1

This appendix formed part of the original submission and has been peer reviewed. We post it as supplied by the authors.

Supplement to: Pardiñas AF, Kappel DB, Roberts M, et al. Pharmacokinetics and pharmacogenomics of clozapine in an ancestrally diverse sample: a longitudinal analysis and genome-wide association study using UK clinical monitoring data. *Lancet Psychiatry* 2023; **10:** 209–19.

PHARMACOKINETICS AND PHARMACOGENOMICS OF CLOZAPINE IN AN ANCESTRALLY DIVERSE SAMPLE: A LONGITUDINAL ANALYSIS AND GENOME-WIDE ASSOCIATION STUDY USING UK CLINICAL MONITORING DATA

APPENDIX 1

Supplementary Methods 2

Curation of the pharmacokinetic data 2

Curation of the genomic data 2

Prediction of biogeographic genomic ancestry 3

GWAS model fitting and statistical fine-mapping 4

Estimation of SNP-based heritability 6

Polygenic score association analysis 6

Supplementary Results 7

Statistical fine-mapping of GWAS loci 7

Support for SNPs previously associated to clozapine metabolism through GWAS 7

Ancestry-specific effect sizes of main GWAS SNPs 8

Heritability and polygenicity of clozapine metabolism 8

Supplementary Table 2 9

Supplementary Table 3 10

Supplementary Table 7 11

Supplementary Table 8 12

Supplementary Figure 1 13

Supplementary Figure 2 14

Supplementary Figure 3 15

Supplementary Figure 4 16

Supplementary Figure 5 17

Supplementary Figure 6 18

Supplementary References 19

Supplementary Methods

Curation of the pharmacokinetic data

19,096 pharmacokinetic assays were available for 4,760 CLOZUK individuals, including information on the clozapine and norclozapine plasma concentrations, the daily clozapine dose, and the time of both the drug intake and blood draw. All plasma concentrations were determined by a standard high-performance liquid chromatography mass spectrometry (HPLC-MS) procedure at Magna Laboratories (Ross-on-Wye, U.K.), and further details are provided in a previous publication [1]. The pharmacokinetic assay dataset was assessed for potential clerical errors (e.g. negative time periods, multiple samples recorded at the same time point) and cross-checked with ZTAS records to ensure data integrity (e.g. correct year of birth for each individual). Data from all assays containing any erroneous information was removed. Additionally, assays were excluded from further analyses if they fitted any of the following criteria: (i) blood drawn outside of a "trough sample" interval of 6- to 24-hours postdose [2]; (ii) clozapine or norclozapine concentration <50 ng/mL, outside the minimum detection range of the HPLC-MS instrument; (iii) clozapine concentration >2000 ng/mL, reaching the range of potential toxicity [3]; (iv) clozapine:norclozapine ratio outside of the 0.5-3.0 interval, indicating potential non-adherence to treatment [4]; and (v) clozapine dose >900 mg/day, the maximum advised by the British National Formulary. Finally, due to potentially different treatment regimes, we also removed all data from individuals under 18 years of age.

Curation of the genomic data

The CLOZUK2 samples underwent genotyping, quality control and imputation as previously described [1]. Briefly, 7,417 samples were genotyped by deCODE Genetics (Reykjavík, Iceland), using an Illumina HumanOmniExpress-12 array with 719,665 SNPs. Quality control was performed using PLINK v1.9 [5]. Samples and markers with a missingness rate of >2% and samples with an inbreeding coefficient of F>0.2 were excluded from further analyses. After curation and merging with the pharmacokinetic assay dataset, 3,578 samples genotyped at 698,442 SNPs remained in CLOZUK2.

For CLOZUK3, 1,439 samples were genotyped at the Icahn School of Medicine at Mount Sinai (New York City, USA) using an Illumina® Infinium Global Screening Array-24 (GSA-24) with 654,027 SNPs. The curation of both samples and markers was performed using the same procedures as CLOZUK2, implemented in the DRAGON-Data pipeline [6]. After curation and

merging with the pharmacokinetic assay dataset, 917 samples genotyped at 537,334 SNPs remained in CLOZUK3.

Genotype imputation for both cohorts was performed using the Haplotype Reference Consortium (HRC) panel through the Michigan Imputation Server [7]. All imputed genotype dosages of CLOZUK2 and CLOZUK3 were further curated within each cohort with the following parameters: Imputation quality $r^2 \geq 0.7$; hard-call genotype probability ≥ 80%; hard-call missingness ≤ 5%; minor allele count (MAC) ≥ 2. The remaining variants in common between both samples were then merged and a second round of dosage curation was performed with the following parameters: Hard-call missingness ≤ 2%; MAC ≥ 400 and Hardy-Weinberg equilibrium (HWE) mid-p > 10^{-6}. This led to over 2.9 million high-quality markers for GWAS and polygenic risk score (PRS) analyses. For context, the choice of setting quality control thresholds on MAC instead of minor allele frequency (MAF) was motivated by evidence relating this parameter to the power and robustness of multiple genomic association tests [8, 9], for which usual MAF thresholds are not always relevant.

Prediction of biogeographic genomic ancestry

Genomic data was also used for biogeographic ancestry prediction using a linear discriminant analysis (LDA) model based on ancestry-informative markers [AIMs; 10]. Briefly, genotype data was merged with a public geo-localised reference panel based on the Human Genome Diversity Project [HGDP; 11]. The population differentiation statistic F_{ST} was used to identify AIMs by selecting those overlapping SNPs maximally differentiated between HGDP superpopulations. Genotype principal components (PCs) were then generated based on these AIMs and processed through a LDA model with the HGDP samples of known ancestry as training set and CLOZUK2/CLOZUK3 as test sets. The model resulted in a set of probabilities, for each sample, of belonging to each of five global biogeographic ancestries: “European”, “North African”, “Sub-Saharan African”, “East Asian” and “Southwest Asian” [10]. For comparability with other studies, it should be noted that these ancestries are equivalent in definition to the non-admixed standardised biogeographic groups recently proposed for use in pharmacogenomics research by Huddart et al. [12]. Individuals were assigned to one specific ancestry if their LDA probability surpassed a threshold of 80%, and to a group labelled as “admixed/unknown” otherwise. For carrying out ancestry-specific GWAS, given the small sample size of some of these subgroups, the merged CLOZUK dosages were curated within

each ancestry with the following parameters: Hard-call missingness ≤ 2%; hard-call MAC ≥ 40 and HWE mid-p > 10^{-4}.

GWAS model fitting and statistical fine-mapping

The equation of a generalised linear mixed model (GLMM), as used for pharmacokinetic, GWAS, and PRS association tests, takes the following form in matrix notation [13]:

$$y = X\beta + Z\gamma + e$$

Where y is the outcome variable, in our case the plasma concentrations of either clozapine or norclozapine, or the clozapine:norclozapine metabolic ratio. X is a matrix of variables with a corresponding matrix β of fixed effects, in our case formed by genetic (SNPs/PRSs, PCs, ancestry probabilities) and non-genetic predictors (sex, age, clozapine dose, time between dose intake and blood sample). Z is a matrix of grouping variables with a corresponding matrix γ of random effects, in our case a CLOZUK sample ID which uniquely identifies all pharmacokinetic assays from the same person, Finally, e is an error term of unexplained ("residual") variability.

For our main GWAS analyses, we estimated effect sizes, standard errors and p-values for each SNP using the Wald test implemented in TrajGWAS. A thorough description of this method, including power curves for a range of variant effect sizes and MAFs can be found in Ko et al. [14]. TrajGWAS uses the "within-subject variance estimator by robust regression" (WiSER) approach [15], a GLMM framework that can accommodate predictors of the outcome mean and variance while being robust to multiple outcome and random effect distributions. However, computational problems with the WiSER procedure may occur while modelling long-tailed outcome distributions, requiring changing the numerical optimisation procedures for particular genomic regions or SNPs [14]. As long tails and extreme values are often features of pharmacokinetic metrics [16], we sought to avoid this potential problem by normalising and standardising all of our outcomes before the TrajGWAS analysis. For this we used a cube root transformation for the gamma-distributed clozapine and norclozapine concentrations [17, 18], and a logarithmic transformation for the log-normally-distributed metabolic ratio. All covariates were also standardised, as recommended to improve the computational performance of GLMMs [19]. These procedures were also used in the genomic *glmmTMB* analyses.

Taking advantage of the WiSER modelling capabilities, TrajGWAS models included sex as a predictor of both the between-person mean and the within-person variance, reflecting the

observation that differential smoking habits in males and females might account for some of the "noise" in clozapine pharmacokinetic assays [20]. When replicating the results of specific SNPs in *glmmTMB* (**Supplementary Table 3**), we approximated this approach by setting sex as a predictor of both the mean ("location") and the residual variance ("scale") in the GLMM. We also included a second-order polynomial term (age^2) among our fixed effects in all models. This is a standard way of modelling non-linear relationships between outcomes and predictors in linear models [21], in this case reflecting the potentially steeper change in drug pharmacokinetics that may occur with aging [22].

By implementing a generic SNP-level association test to longitudinal genomic datasets, trajGWAS can be employed in both ancestry-specific and cross-ancestry experimental designs. It has been shown that GWAS approaches employing the cross-ancestry framework can achieve greater discovery power than ancestry-specific studies by avoiding the exclusion of admixed individuals [23], as well as by taking advantage of similarities in variant effect size across ancestries [24]. However, the greater diversity in cross-ancestry samples requires a tighter control of population stratification to prevent genomic inflation, which was addressed in our main analysis by covarying for 10 PCs and 4 biogeographical ancestry LDA probabilities. These are two related but not redundant measures of "global" genetic ancestry [24], with the latter being a quantitative approximation to the ancestral background of the individual that might incorporate non-genetic (cultural or environmental) diversity relevant to the phenotype under study, as well as genetic information not captured by the top PCs [25]. It should be noted that trajGWAS, although made for fitting GLMMs to genetic data, does not incorporate an option for using a kinship or genetic relatedness matrix (GRM) as the covariance structure of its individual-level random effect term to account for ancestry, cryptic relatedness or family structure. That function is characteristic of other statistical genomics software derived from the "variance components" GLMM framework [26], such as GCTA [27] or GENESIS [28]. These were not designed for fitting complex regression models to longitudinal data and carrying out the adaptations and tests required for this purpose was beyond the scope of our study. The use of variance components GLMMs in cross-sectional datasets with population stratification has been reviewed elsewhere [29].

Secondary ancestry-specific GWAS were carried out using the saddlepoint approximation (SPA) to the score test also implemented in TrajGWAS, which is appropriate even for small sample sizes and rare allele frequencies [14]. These analyses did not include ancestry probabilities as covariates, though still included the full set of 10 PCs to control for population

stratification. Ancestry-specific GWAS were not run in the sample of individuals classified as "admixed/unknown" due to their potential ancestral complexity; nor in those of East Asian ancestry due to the small sample size of this group (n=41).

Genomic inflation statistics (λ values) were calculated on the output of each GWAS using the *fastman* R package [30]. LD clumping was also performed to summarise the results and identify tagged genes. LD clumps were formed around genome-wide significant ("index") SNPs and variants with r^2>0.1 within 3000 kb were assigned to these clumps. Fine-mapping of genome-wide significant clumps was carried out with FINEMAP v1.41 [31], setting the maximum number of causal SNPs ("k") parameter to k=5, and employing locus-wide LD panels created from the CLOZUK dosage data. Clumps in common between different outcomes were additionally run through flashfm v1.0 [32] using the top 1,000 causal configurations identified by FINEMAP, in order to pinpoint potentially shared causal SNPs. Additionally, for each genome-wide significant GWAS locus and phenotype, dosages from the fine-mapped SNP with the largest probability of being causal were extracted and analysed in R (using *glmmTMB*) to estimate GLMM effect sizes in the scale of the original pharmacokinetic variables.

Estimation of SNP-based heritability

We used MiXeR v1.3 to estimate the heritability of clozapine metabolism phenotypes using summary statistics, given its reported good performance across a range of genomic architectures including oligogenic traits [33]. An LD reference panel was generated directly from CLOZUK best-guess genotypes and all analyses were ran using default parameters. As recommended by the software developers, results were generated from 20 MiXeR runs using random sets of ~500,000 LD-independent SNPs (MAF > 0.05; $r^2 < 0.9$).

Polygenic score association analysis

As described in the main text, GLMM regression models to estimate PRS effect sizes followed the main pharmacokinetic analyses. As an index of the proportion of variance explained by PRS in the context of other fixed and random effects, a semi-partial R^2 statistic was estimated in R using *partR2* [34]. Given the current implementation of this method cannot accommodate gamma-distributed GLMMs, all PRS models were re-fitted for R^2 estimation using normalised phenotypes. Following a recent recommendation by Rights and Sterba [35], time-varying fixed-effect predictors (age, age^2, clozapine daily dose and TDS) were also centred at the individual level ("de-meaned") in the re-fitted LMM models.

Supplementary Results

Statistical fine-mapping of GWAS loci

The best-fitting FINEMAP models for *CYP1A1/1A2* and *POR* included just a single causal variant (k=1); while *UGT1A**, *UGT2B10* (in norclozapine) and *CYP2C18* had two (k=2). The metabolic ratio locus at *UGT2B10* could not be confidently fine-mapped with the default software settings (best-fitting k ≥ 5). We reran FINEMAP at this locus using a conditional analysis approach, which inferred a best causal model with k=7. Based on the data from the best FINEMAP model for each locus, we then defined credible sets with 95% posterior probability of including all causal SNPs at a locus. These contained between 1 and 411 variants, each with an estimated PPI value indicating its probability of being a causal variant (**Supplementary Table 5**). Apart from the metabolic ratio *CYP2C18* locus, all FINEMAP credible sets contained fewer SNPs than a previous fine-mapping effort [1]. Within credible sets, one SNP was confidently identified as the only causal signal behind the *CYP1A1/1A2* locus for both clozapine and norclozapine (rs2472297, PPI=1). SNPs with high causal probabilities (PPI ≥ 0.33) were also found within the boundaries of two genes: *UGT2B10* (norclozapine and metabolic ratio) and *POR* (metabolic ratio). These genes themselves cumulatively contained most of the PPI within each locus, suggesting they are the most likely genes in the region to be causally related to the association signal [36].

Support for SNPs previously associated to clozapine metabolism through GWAS

Several variants have been highlighted before as putative pharmacogenomic markers of clozapine metabolism in European ancestry studies [1, 37]. These include a SNP intergenic to *CYP1A1/CYP1A2* (rs2472297), fine-mapped missense variants in *UGT2B10* (rs61750900), *UGT1A4* (rs2011425) and *CYP2C18* (rs1126545), and an intronic eQTL in *NFIB* (rs28379954). From this list, our credible sets defined by FINEMAP and flashfm included rs2472297 (largest PPI=1), rs2011425 (largest PPI=0.026) and rs1126545 (largest PPI=0.021). The other SNPs were not found within these results. On the *UGT1A4* locus, we instead found several other missense SNPs within the FINEMAP credible sets, but the majority were within a *UGT1A5* exon (4/8 for clozapine, cumulative PPI=0.119; 4/7 for norclozapine, cumulative PPI=0.110). This result was consistent with the smaller flashfm credible sets, which had most of their missense variants also mapped to *UGT1A5* (5/6 for clozapine, cumulative PPI=0.127; 5/6 for norclozapine, cumulative PPI=0.120). On the *UGT2B10* locus, for either norclozapine or the metabolic ratio, none of our analyses included any missense variants as part of credible

sets, and in fact those were largely different between the assessed phenotypes. It should be noted this result could have been confounded by the large number of causal SNPs inferred for the metabolic ratio GWAS, which itself might have arisen due to the complexity and breadth of the association signal (1385 SNPs). Finally, rs28379954 could not be evaluated in our GWAS due to the poor imputation quality of the *NFIB* gene within CLOZUK2 and CLOZUK3 (imputation $r^2 < 0.6$).

Additional analyses using flashfm were undertaken for loci shared between multiple phenotypes. This led to further shrinking in the *UGT1A** credible set, from 90-107 SNPs in the norclozapine and clozapine analyses, respectively, to 74 SNPs (**Supplementary Table 5**). However, the credible sets of *CYP1A2* and *UGT2B10* widened, likely because flashfm uses information from all the FINEMAP models, not just those that are the best-fit, and thus reflects uncertainty in the number of causal variants underpinning the GWAS signal. Using flashfm also revealed a set of SNPs in high LD (r^2>0.7) within the *UGT2B10* locus that were putatively causal for both phenotypes and partially within the coding region of this gene (SNP group “A”; norclozapine cumulative PPI=0.169; ratio cumulative PPI=1; **Supplementary Table 5**).

Ancestry-specific effect sizes of main GWAS SNPs

The cross-ancestry consistency of the markers in **Table 4** was explored by re-fitting the regression models within the CLOZUK ancestry groups. Across 40 tests (10 SNPs x 4 ancestries), we saw consistency (in direction and magnitude) of all SNP effect sizes with the main analysis in 38 instances, including every comparison in which the ancestry-specific association statistics were at least nominally significant (**Supplementary Table 6**).

Heritability and polygenicity of clozapine metabolism

The univariate analyses models implemented in MiXeR supported the oligogenicity of clozapine metabolism, with less than 0.001% of genetic variants genome-wide having non-zero effects for any of the analysed phenotypes. Common SNP-based heritability values were estimated at 2.87% (SE=0.30%) for clozapine; 4.86% (SE=0.28%) for norclozapine and 8.62% (SE=0.59%) for the metabolic ratio. Despite these low values, all the model fitting performance metrics (Akaike’s Information Criterion, AIC; Bayesian Information Criterion, BIC) for these models were positive with the only exception of the BIC value of clozapine, supporting that the current TrajGWAS analysis is sufficiently powered for this procedure.

Supplementary Table 2

Additional fixed-effect covariates for the four clozapine pharmacokinetics models reported in **Table 2**, as estimated with GLMM regression. Predictor names indicate their unit of measurement or, in the case of binary predictors, their non-reference level. Effect sizes (β) indicate the positive or negative impact of a one-unit increase of the predictor in the average of the outcome, across all the individuals and longitudinal assays of the cross-ancestry CLOZUK sample.

Predictor	Log(dose)			Clozapine			Norclozapine			Log(Metabolic Ratio)		
	β	SE	p	β	SE	p	β	SE	p	β	SE	p
Daily dose (mg/day)	-	-	-	0.002	$3.60x10^{-5}$	$4.02x10^{-426}$	$6.01x10^{-4}$	$2.13x10^{-5}$	$5.26x10^{-175}$	$-8.12x10^{-5}$	$1.82x10^{-5}$	$8.01x10^{-6}$
Time between dose and blood draw (hours)	-0.003	0.0010	0.009	-0.010	0.0015	$4.77x10^{-10}$	0.005	0.0009	$7.64x10^{-9}$	-0.010	0.0008	$2.94x10^{-34}$
Sex (male)	0.129	0.0137	$8.35x10^{-21}$	-0.147	0.0179	$1.58x10^{-16}$	-0.032	0.0096	$7.87x10^{-4}$	-0.015	0.0089	0.102
Age (years)	-0.001	0.0005	0.051	0.004	0.0007	$1.34x10^{-9}$	$9.77x10^{-4}$	0.0004	0.010	$7.09x10^{-4}$	0.0004	0.043
Age^2 ($years^2$)	$-2.93x10^{-4}$	$3.45x10^{-5}$	$2.14x10^{-17}$	$6.61x10^{-6}$	$4.60x10^{-5}$	0.886	$4.24x10^{-5}$	$2.50x10^{-5}$	0.090	$-3.22x10^{-5}$	$2.30x10^{-5}$	0.161
Batch (CLOZUK3)	-0.060	0.0148	$4.78x10^{-5}$	0.022	0.0190	0.236	0.032	0.0102	0.002	-0.017	0.0094	0.065

Supplementary Table 3

Effect sizes of fine-mapped genome-wide significant SNPs associated with clozapine metabolism phenotypes, in a ng/mL scale for plasma concentrations and a log scale for the ratio. PPI: Largest posterior probability of the variant being causal across FINEMAP and flashfm analyses. Unadjusted effect size estimates, controlled only for genomic covariates and a random effect, are given in **Supplementary Table 7**.

SNP	Phenotype	Closest gene(s)	Effect allele	Other allele	Effect allele frequency	PPI	TrajGWAS	glmmTMB		
							p	β	SE	p
**rs3732218**	Clozapine	*UGT1A5*<br>*UGT1A6*<br>*UGT1A7*<br>*UGT1A8*<br>*UGT1A9*<br>*UGT1A10*	A	G	9.99%	8.61%	3.26×10^{-12}	-0.125	0.0183	9.17×10^{-12}
**rs2472297**	Clozapine	*CYP1A1*<br>*CYP1A2*	T	C	23.91%	100%	4.39×10^{-11}	-0.086	0.0132	7.60×10^{-11}
**rs2926036**	Norclozapine	*UGT2B10*	G	A	85.51%	34.89%	3.16×10^{-28}	0.165	0.0160	4.99×10^{-25}
**rs115619871***	Norclozapine	*UGT2B10*	T	C	9.82%	6.71%	2.50×10^{-19}	-0.148	0.0173	1.26×10^{-17}
**rs3732218**	Norclozapine	*UGT1A5*<br>*UGT1A6*<br>*UGT1A7*<br>*UGT1A8*<br>*UGT1A9*<br>*UGT1A10*	A	G	9.99%	10.77%	9.36×10^{-13}	-0.120	0.0172	2.66×10^{-12}
**rs2472297**	Norclozapine	*CYP1A1*<br>*CYP1A2*	T	C	23.91%	100%	2.10×10^{-9}	-0.075	0.0124	1.04×10^{-9}
**rs1902932**	Ratio	*UGT2B10*	A	G	9.84%	40.12%	1.32×10^{-123}	0.171	0.0089	2.44×10^{-83}
**rs115619871***	Ratio	*UGT2B10*	T	C	9.82%	36.88%	2.30×10^{-123}	0.171	0.0089	4.33×10^{-83}
**rs76413136**	Ratio	*CYP2C18*	T	C	23.25%	10.60%	9.18×10^{-22}	0.060	0.0065	2.75×10^{-20}
**rs41301394**	Ratio	*POR*	T	C	26.79%	43.68%	4.81×10^{-8}	-0.034	0.0061	2.27×10^{-8}

* Putatively shared causal variant for norclozapine and the clozapine:norclozapine metabolic ratio reported by flashfm.

Supplementary Table 7

Pharmacokinetic outcomes stratified by genotype of genome-wide significant SNPs associated with clozapine metabolism phenotypes. To account for both longitudinal measurements and covariates, intercept-free GLMMs were used to calculate outcome means and standard errors, as suggested by Schielzeth [21]. Scales used are ng/mL for plasma concentrations and a natural scale for the clozapine:norclozapine ratio.

SNP	Phenotype	Closest gene(s)	Effect allele	Other allele	OA/OA		EA/OA		EA/EA	
					Mean	SE	Mean	SE	Mean	SE
**rs3732218**	Clozapine	*UGT1A5* *UGT1A6* *UGT1A7* *UGT1A8* *UGT1A9* *UGT1A10*	A	G	0.542	0.008	0.474	0.011	0.461	0.036
**rs2472297**	Clozapine	*CYP1A1* *CYP1A2*	T	C	0.551	0.009	0.504	0.009	0.467	0.016
**rs2926036**	Norclozapine	*UGT2B10*	G	A	0.207	0.010	0.262	0.005	0.305	0.004
**rs115619871***	Norclozapine	*UGT2B10*	T	C	0.299	0.004	0.262	0.005	0.192	0.015
**rs3732218**	Norclozapine	*UGT1A5* *UGT1A6* *UGT1A7* *UGT1A8* *UGT1A9* *UGT1A10*	A	G	0.298	0.004	0.264	0.006	0.237	0.017
**rs2472297**	Norclozapine	*CYP1A1* *CYP1A2*	T	C	0.302	0.005	0.280	0.005	0.261	0.008
**rs1902932**	Ratio	*UGT2B10*	A	G	1.823	0.013	2.121	0.019	2.498	0.073
**rs115619871***	Ratio	*UGT2B10*	T	C	1.822	0.013	2.121	0.019	2.497	0.073
**rs76413136**	Ratio	*CYP2C18*	T	C	1.836	0.014	1.933	0.016	2.068	0.032
**rs41301394**	Ratio	*POR*	T	C	1.911	0.015	1.858	0.016	1.796	0.027

* Putatively shared causal variant for norclozapine and the metabolic ratio reported by flashfm.

Supplementary Table 8

Effect sizes of fine-mapped genome-wide significant SNPs associated with clozapine metabolism phenotypes, in a ng/mL scale for plasma concentrations and a log scale for the ratio. Only genomic covariates (principal components and ancestry probabilities) and an individual-level random effect have been included in these models. PPI: Largest posterior probability of the variant being causal across FINEMAP and flashfm.

SNP	Phenotype	Closest gene(s)	Effect allele	Other allele	Effect allele frequency	PPI	TrajGWAS	glmmTMB		
							p	β	SE	p
**rs3732218**	Clozapine	*UGT1A5*<br>*UGT1A6*<br>*UGT1A7*<br>*UGT1A8*<br>*UGT1A9*<br>*UGT1A10*	A	G	9.99%	8.61%	3.26×10^{-12}	-0.085	0.0183	3.17×10^{-6}
**rs2472297**	Clozapine	*CYP1A1*<br>*CYP1A2*	T	C	23.91%	100%	4.39×10^{-11}	-0.066	0.0132	5.52×10^{-7}
**rs2926036**	Norclozapine	*UGT2B10*	G	A	85.51%	34.89%	3.16×10^{-28}	0.159	0.0165	6.61×10^{-22}
**rs115619871***	Norclozapine	*UGT2B10*	T	C	9.82%	6.71%	2.50×10^{-19}	-0.146	0.0179	3.39×10^{-16}
**rs3732218**	Norclozapine	*UGT1A5*<br>*UGT1A6*<br>*UGT1A7*<br>*UGT1A8*<br>*UGT1A9*<br>*UGT1A10*	A	G	9.99%	10.77%	9.36×10^{-13}	-0.078	0.0177	1.02×10^{-5}
**rs2472297**	Norclozapine	*CYP1A1*<br>*CYP1A2*	T	C	23.91%	100%	2.10×10^{-9}	-0.055	0.0128	1.57×10^{-5}
**rs1902932**	Ratio	*UGT2B10*	A	G	9.84%	40.12%	1.32×10^{-123}	0.170	0.0089	4.92×10^{-81}
**rs115619871***	Ratio	*UGT2B10*	T	C	9.82%	36.88%	2.30×10^{-123}	0.170	0.0089	8.25×10^{-81}
**rs76413136**	Ratio	*CYP2C18*	T	C	23.25%	10.60%	9.18×10^{-22}	0.059	0.0065	2.29×10^{-19}
**rs41301394**	Ratio	*POR*	T	C	26.79%	43.68%	4.81×10^{-8}	-0.034	0.0061	2.24×10^{-8}

* Putatively shared causal variant for norclozapine and the metabolic ratio reported by flashfm.

Supplementary Figure 1

Manhattan plots of the GWAS analyses of clozapine metabolism carried out in the European subset of CLOZUK. Black horizontal line indicates the genome-wide significant p-value cutoff of $5x10^{-8}$. A: clozapine plasma concentrations (λ_{GC}= 1.015). B: norclozapine plasma concentrations (λ_{GC}= 1.017). C: clozapine:norclozapine metabolic ratio (λ_{GC}= 0.995).

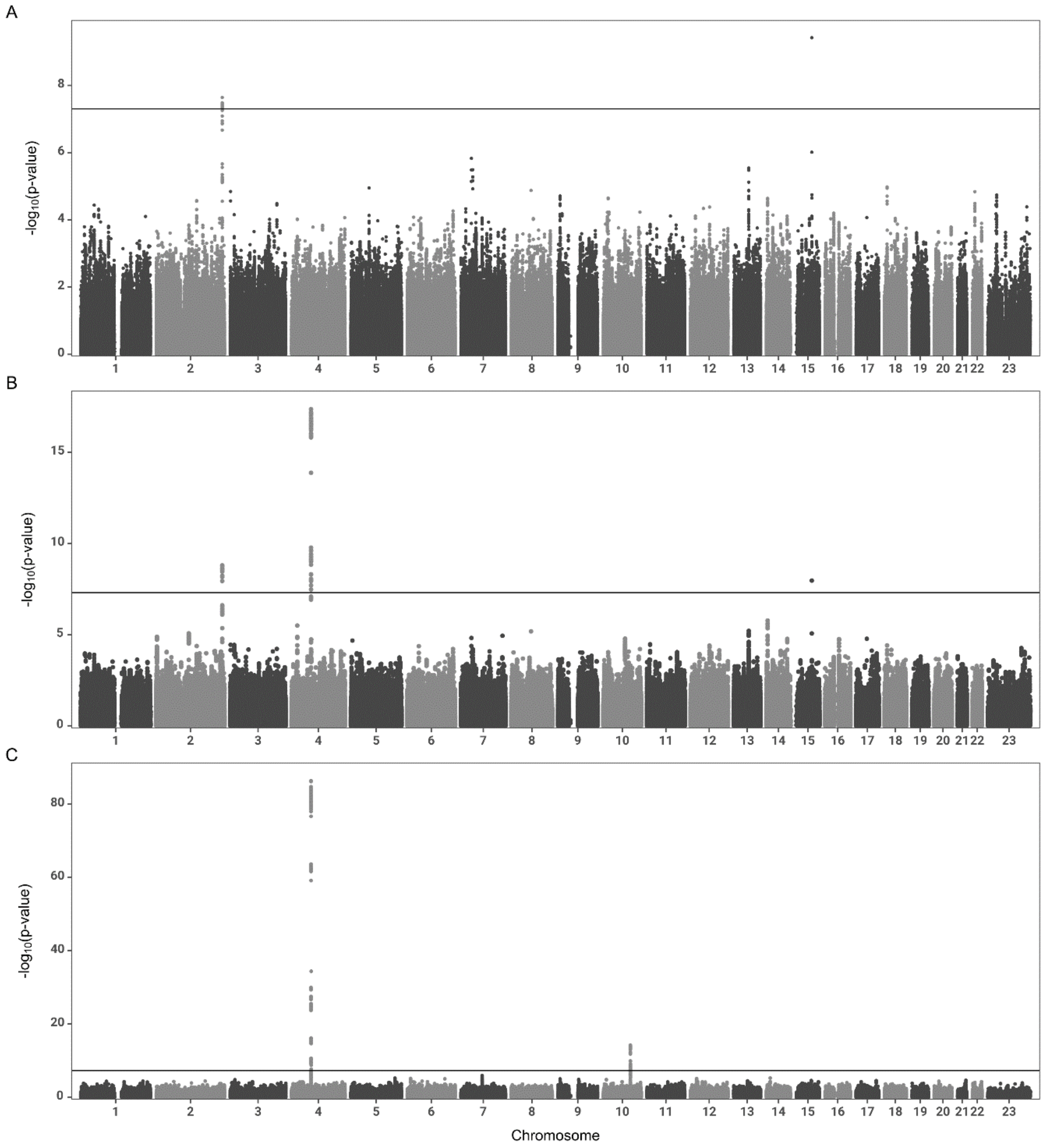

Supplementary Figure 2

Manhattan plots of the GWAS analyses of clozapine metabolism carried out in the Sub-Saharan African subset of CLOZUK. Black horizontal line indicates the genome-wide significant p-value cutoff of $5x10^{-8}$. A: clozapine plasma concentrations (λ_{GC}= 1.066). B: norclozapine plasma concentrations (λ_{GC}= 1.066). C: clozapine:norclozapine metabolic ratio (λ_{GC}= 1.082).

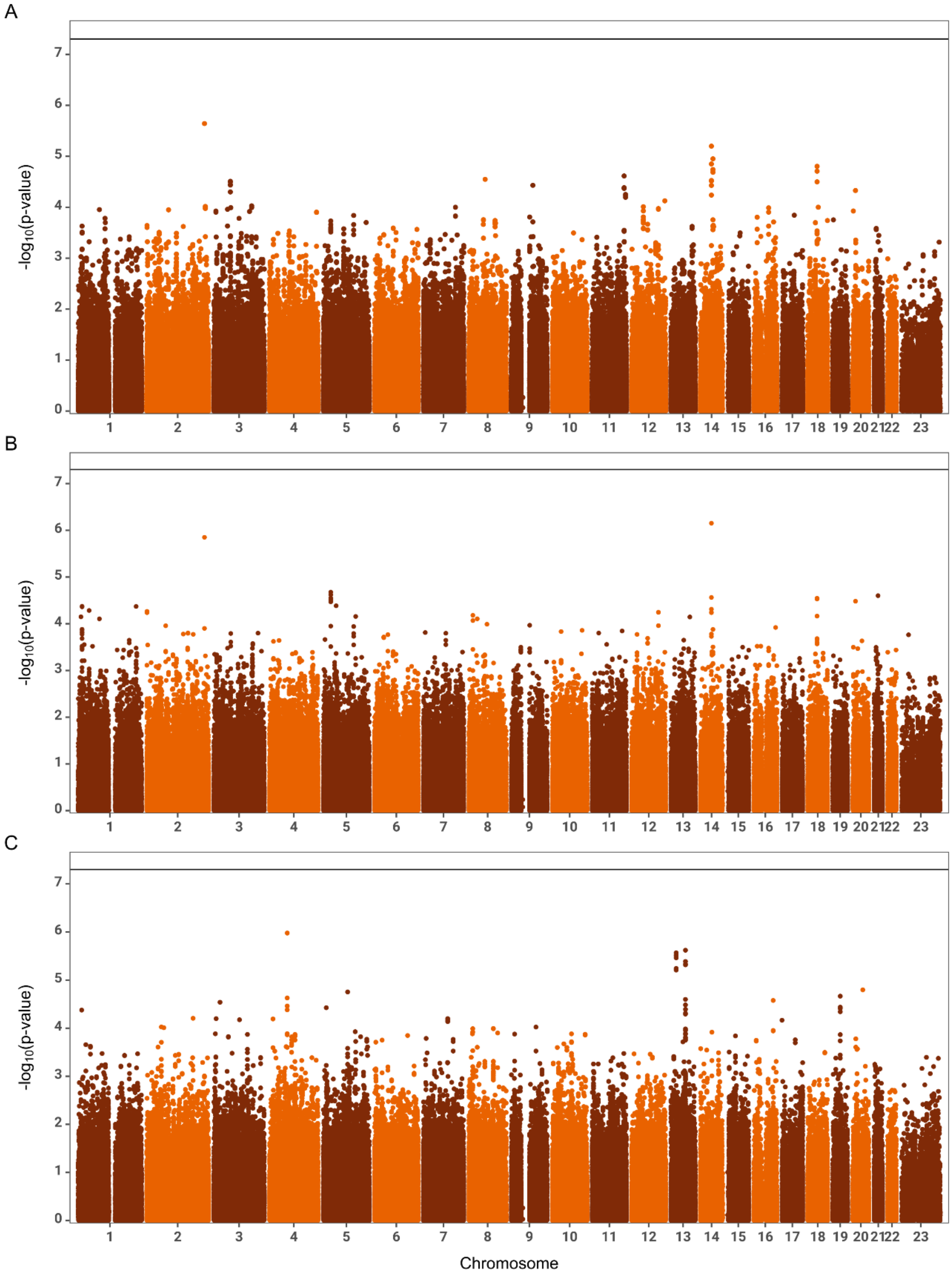

Supplementary Figure 3

Manhattan plots of the GWAS analyses of clozapine metabolism carried out in the North African subset of CLOZUK. Black horizontal line indicates the genome-wide significant p-value cutoff of $5x10^{-8}$. A: clozapine plasma concentrations (λ_{GC}= 1.171). B: norclozapine plasma concentrations (λ_{GC}= 1.072). C: clozapine:norclozapine metabolic ratio (λ_{GC}= 1.031).

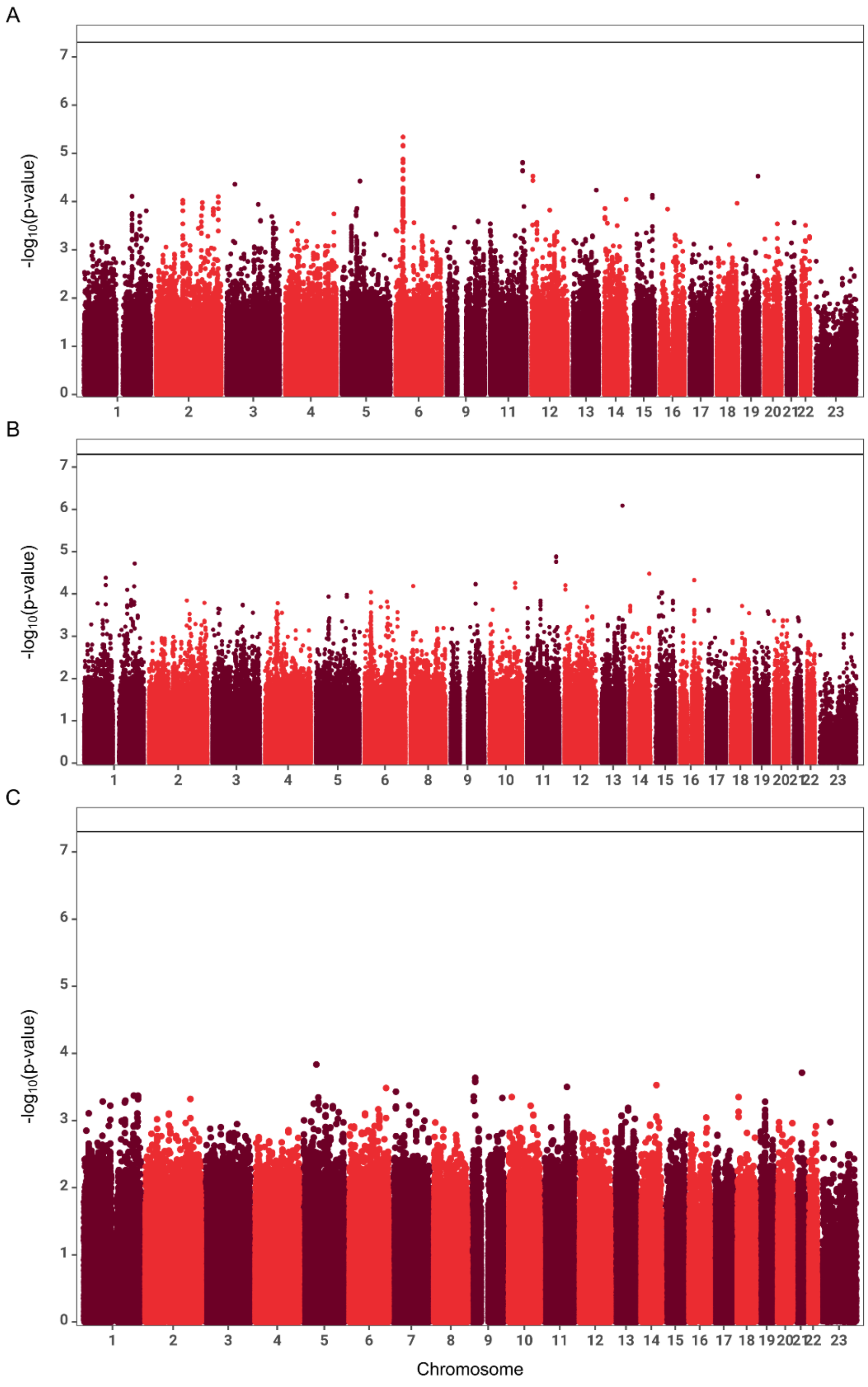

Supplementary Figure 4

Manhattan plots of the GWAS analyses of clozapine metabolism carried out in the Southwest Asian subset of CLOZUK. Black horizontal line indicates the genome-wide significant p-value cutoff of $5x10^{-8}$. A: clozapine plasma concentrations (λ_{GC}= 1.073). B: norclozapine plasma concentrations (λ_{GC}= 1.076). C: clozapine:norclozapine metabolic ratio (λ_{GC}= 1.084).

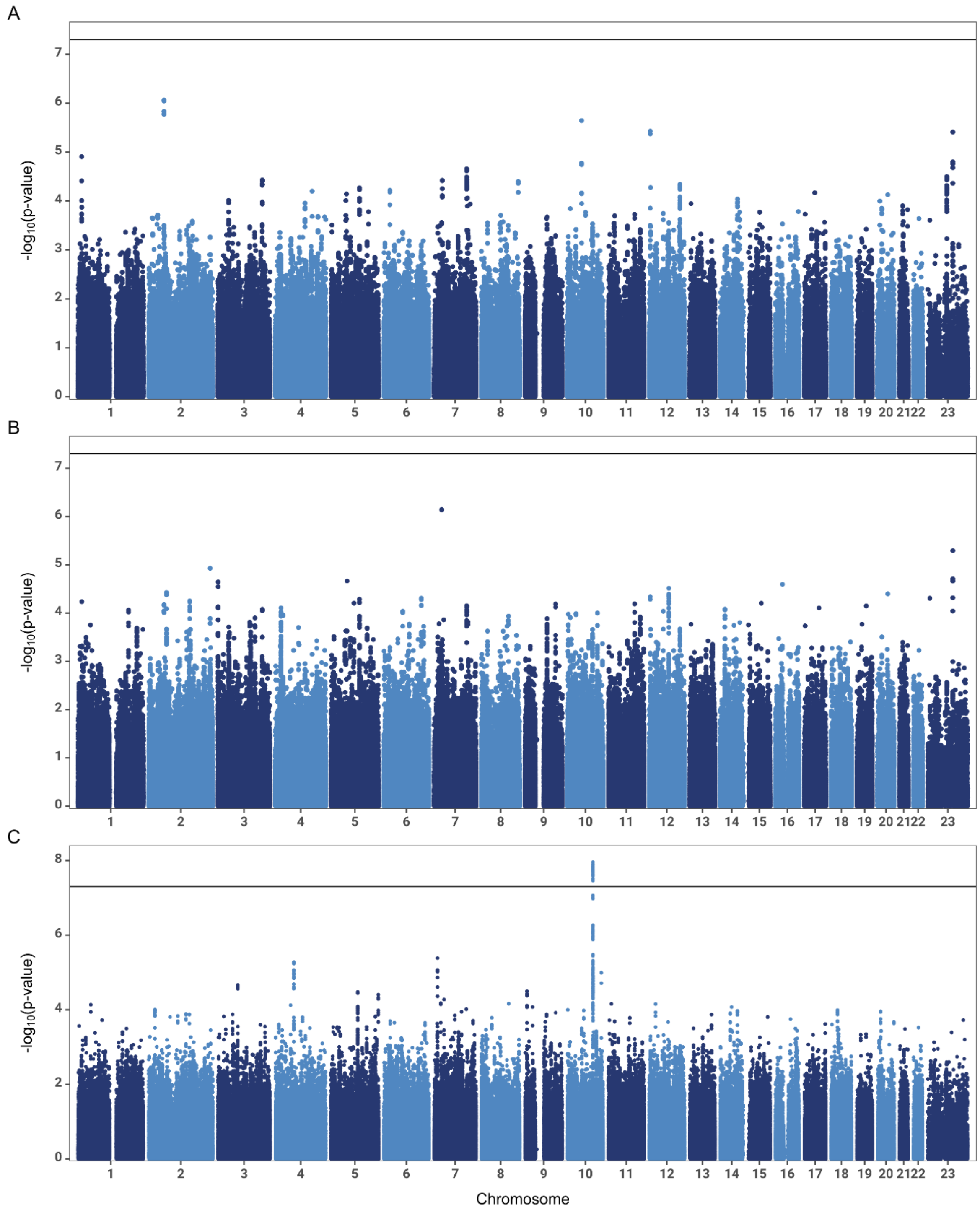

Supplementary Figure 5

Association between polygenic scores for clozapine metabolism generated in CLOZUK2 and their corresponding phenotypes in the Sub-Saharan African and Southwest Asian subsets of CLOZUK3. Dots and whiskers indicate the estimated value of the regression effect size and its corresponding 95% confidence interval. Bold colours indicate nominally significant associations; semi-transparent colours indicate non-significant effect sizes.

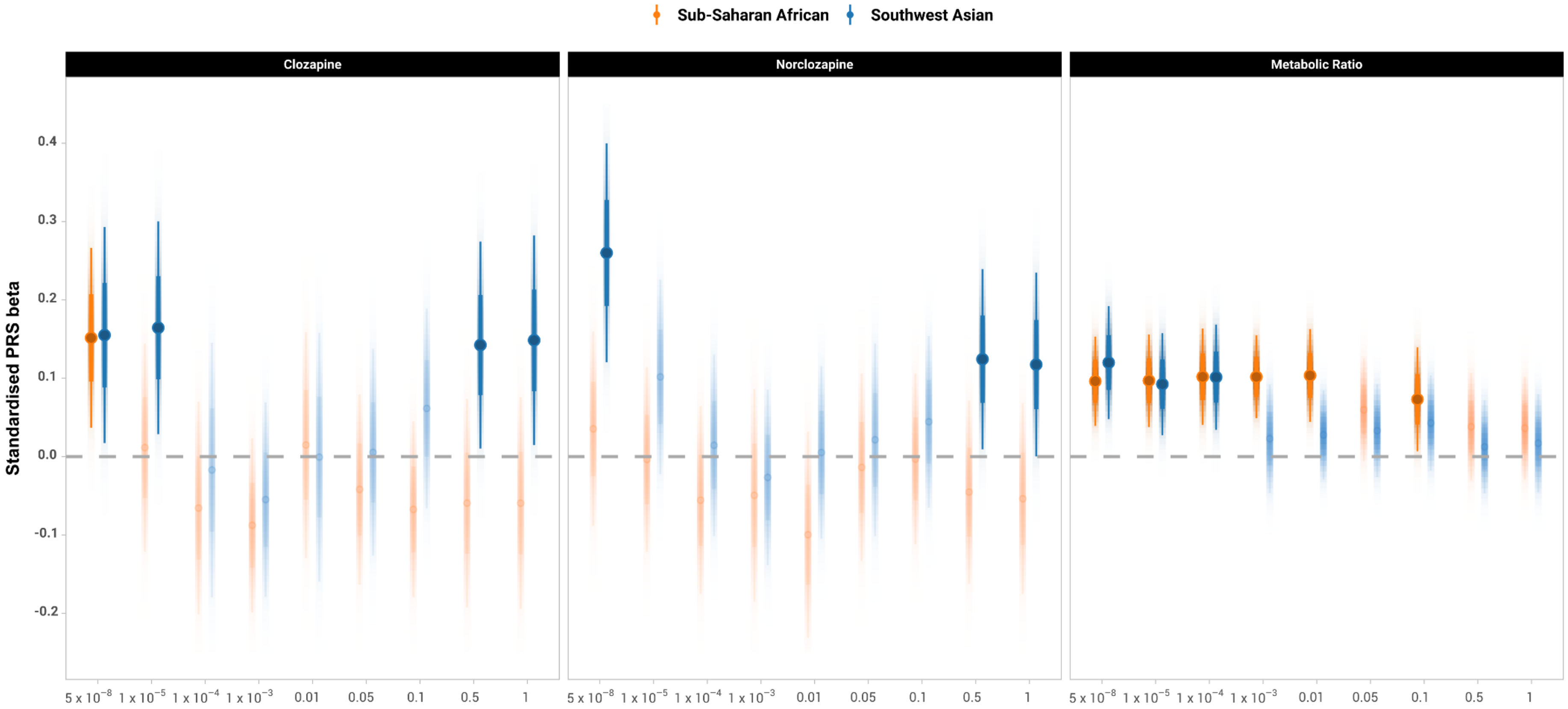

Supplementary Figure 6

Full version of **Figure 1C**. Marginal effects of the ancestry groups in the relationship between clozapine doses and the probability of reaching clozapine levels inside or outside the therapeutic range (350-600 ng/mL). Shaded areas in the probability lines highlight a 95% confidence interval. Vertical dashed bars highlight the doses required by individuals in each ancestry group to reach the therapeutic range with 50% probability.

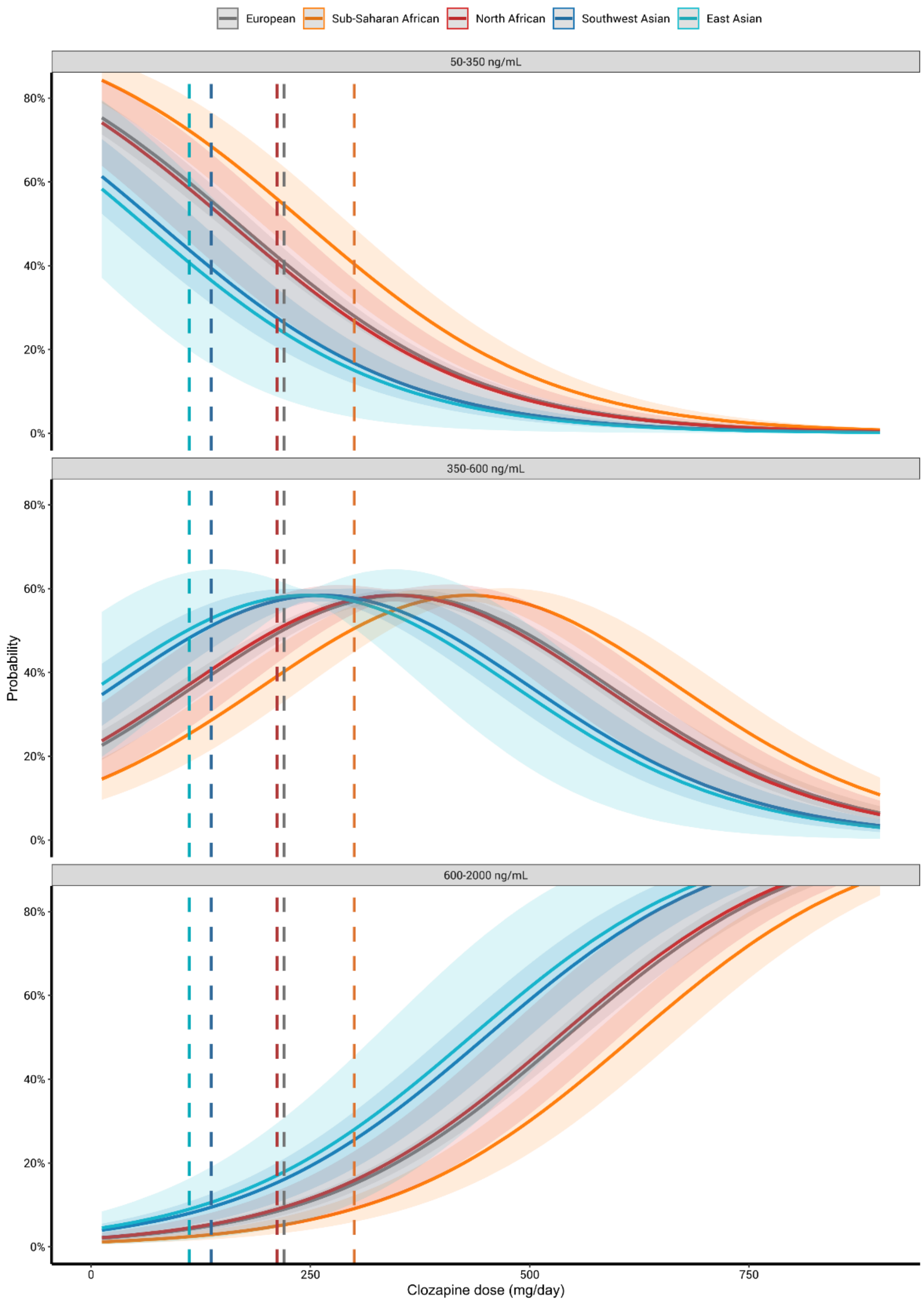

Supplementary References

1. Pardiñas AF, Nalmpanti M, Pocklington AJ, Legge SE, Medway C, King A, et al. Pharmacogenomic Variants and Drug Interactions Identified Through the Genetic Analysis of Clozapine Metabolism. American Journal of Psychiatry. 2019;176(6):477-86.

2. Flanagan RJ. A practical approach to clozapine therapeutic drug monitoring. CHMP Bulletin. 2010;2:4-5.

3. Flanagan RJ, Lally J, Gee S, Lyon R, Every-Palmer S. Clozapine in the treatment of refractory schizophrenia: a practical guide for healthcare professionals. British Medical Bulletin. 2020;135(1):73-89.

4. Ellison JC, Dufresne RL. A review of the clinical utility of serum clozapine and norclozapine levels. Mental Health Clinician. 2015;5(2):68-73.

5. Chang CC, Chow CC, Tellier LC, Vattikuti S, Purcell SM, Lee JJ. Second-generation PLINK: rising to the challenge of larger and richer datasets. GigaScience. 2015;4.

6. Hubbard L, Lynham AJ, Knott S, Underwood JFG, Anney R, Bisson JI, et al. DRAGON-Data: A platform and protocol for integrating genomic and phenotypic data across large psychiatric cohorts. medRxiv. 2022:2022.01.18.22269463.

7. McCarthy S, Das S, Kretzschmar W, Delaneau O, Wood AR, Teumer A, et al. A reference panel of 64,976 haplotypes for genotype imputation. Nat Genet. 2016;48(10):1279-83.

8. Ma C, Blackwell T, Boehnke M, Scott LJ. Recommended Joint and Meta-Analysis Strategies for Case-Control Association Testing of Single Low-Count Variants. Genetic Epidemiology. 2013;37(6):539-50.

9. Ray D, Chatterjee N. Effect of non-normality and low count variants on cross-phenotype association tests in GWAS. European Journal of Human Genetics. 2020;28(3):300-12.

10. Legge SE, Pardiñas AF, Helthuis M, Jansen JA, Jollie K, Knapper S, et al. A genome-wide association study in individuals of African ancestry reveals the importance of the Duffy-null genotype in the assessment of clozapine-related neutropenia. Molecular Psychiatry. 2019;24(3):328-37.

11. Li JZ, Absher DM, Tang H, Southwick AM, Casto AM, Ramachandran S, et al. Worldwide Human Relationships Inferred from Genome-Wide Patterns of Variation. Science. 2008;319(5866):1100-4.

12. Huddart R, Fohner AE, Whirl-Carrillo M, Wojcik GL, Gignoux CR, Popejoy AB, et al. Standardized Biogeographic Grouping System for Annotating Populations in Pharmacogenetic Research. Clinical Pharmacology & Therapeutics. 2019;105(5):1256-62.

13. Laird NM, Ware JH. Random-Effects Models for Longitudinal Data. Biometrics. 1982;38(4):963-74.

14. Ko S, German CA, Jensen A, Shen J, Wang A, Mehrotra DV, et al. GWAS of longitudinal trajectories at biobank scale. The American Journal of Human Genetics. 2022;109(3):433-45.

15. German CA, Sinsheimer JS, Zhou J, Zhou H. WiSER: Robust and scalable estimation and inference of within-subject variances from intensive longitudinal data. Biometrics. 2021;[in press].

16. Lindsey JK, Jones B, Jarvis P. Some statistical issues in modelling pharmacokinetic data. Statistics in Medicine. 2001;20(17-18):2775-83.

17. Wilson EB, Hilferty MM. The Distribution of Chi-Square. Proceedings of the National Academy of Sciences. 1931;17(12):684-8.

18. Terrell GR. The Wilson-Hilferty Transformation Is Locally Saddlepoint. Biometrika. 2003;90(2):445-53.

19. Gelman A, Hill J, Vehtari A. Regression and Other Stories. Cambridge: Cambridge University Press; 2020.

20. Diaz FJ, de Leon J, Josiassen RC, Cooper TB, Simpson GM. Plasma clozapine concentration coefficients of variation in a long-term study. Schizophrenia Research. 2005;72(2):131-5.

21. Schielzeth H. Simple means to improve the interpretability of regression coefficients. Methods in Ecology and Evolution. 2010;1(2):103-13.

22. Perucca E. Age-Related Changes in Pharmacokinetics: Predictability and Assessment Methods. International Review of Neurobiology. 81: Academic Press; 2007. p. 183-99.

23. Li YR, Keating BJ. Trans-ethnic genome-wide association studies: advantages and challenges of mapping in diverse populations. Genome Medicine. 2014;6(10):91.

24. Hou K, Bhattacharya A, Mester R, Burch KS, Pasaniuc B. On powerful GWAS in admixed populations. Nature Genetics. 2021;53(12):1631-3.

25. Conomos Matthew P, Laurie Cecelia A, Stilp Adrienne M, Gogarten Stephanie M, McHugh Caitlin P, Nelson Sarah C, et al. Genetic Diversity and Association Studies in US Hispanic/Latino Populations: Applications in the Hispanic Community Health Study/Study of Latinos. The American Journal of Human Genetics. 2016;98(1):165-84.

26. George AW, Visscher PM, Haley CS. Mapping quantitative trait loci in complex pedigrees: a two-step variance component approach. Genetics. 2000;156(4):2081-92.

27. Yang J, Lee SH, Goddard ME, Visscher PM. GCTA: A Tool for Genome-wide Complex Trait Analysis. The American Journal of Human Genetics. 2011;88(1):76-82.

28. Chen H, Wang C, Conomos Matthew P, Stilp Adrienne M, Li Z, Sofer T, et al. Control for Population Structure and Relatedness for Binary Traits in Genetic Association Studies via Logistic Mixed Models. The American Journal of Human Genetics. 2016;98(4):653-66.

29. Sul JH, Martin LS, Eskin E. Population structure in genetic studies: Confounding factors and mixed models. PLOS Genetics. 2018;14(12):e1007309.

30. Paria SS, Rahman SR, Adhikari K. fastman: A fast algorithm for visualizing GWAS results using Manhattan and Q-Q plots. bioRxiv. 2022:2022.04.19.488738.

31. Benner C, Spencer CCA, Havulinna AS, Salomaa V, Ripatti S, Pirinen M. FINEMAP: efficient variable selection using summary data from genome-wide association studies. Bioinformatics. 2016;32(10):1493-501.

32. Hernández N, Soenksen J, Newcombe P, Sandhu M, Barroso I, Wallace C, et al. The flashfm approach for fine-mapping multiple quantitative traits. Nature Communications. 2021;12(1):6147.

33. Frei O, Holland D, Smeland OB, Shadrin AA, Fan CC, Maeland S, et al. Bivariate causal mixture model quantifies polygenic overlap between complex traits beyond genetic correlation. Nature Communications. 2019;10(1):2417.

34. Stoffel MA, Nakagawa S, Schielzeth H. partR2: partitioning R2 in generalized linear mixed models. PeerJ. 2021;9:e11414.

35. Rights JD, Sterba SK. New Recommendations on the Use of R-Squared Differences in Multilevel Model Comparisons. Multivariate Behavioral Research. 2020;55(4):568-99.

36. Trubetskoy V, Pardiñas AF, Qi T, Panagiotaropoulou G, Awasthi S, Bigdeli TB, et al. Mapping genomic loci implicates genes and synaptic biology in schizophrenia. Nature. 2022;604(7906):502-8.

37. Smith RL, O'Connell K, Athanasiu L, Djurovic S, Kringen MK, Andreassen OA, et al. Identification of a novel polymorphism associated with reduced clozapine concentration in schizophrenia patients—a genome-wide association study adjusting for smoking habits. Translational Psychiatry. 2020;10(1):198.
